# Unloading using Impella CP during profound cardiogenic shock caused by left ventricular failure in a large animal model: impact on the right ventricle

**DOI:** 10.1186/s40635-020-00326-y

**Published:** 2020-08-12

**Authors:** Jakob Josiassen, Ole Kristian Lerche Helgestad, Nanne Louise Junker Udesen, Ann Banke, Peter Hartmund Frederiksen, Janus Adler Hyldebrandt, Henrik Schmidt, Lisette Okkels Jensen, Christian Hassager, Hanne Berg Ravn, Jacob E. Møller

**Affiliations:** 1 Department of Cardiology, Copenhagen University Hospital, Rigshospitalet, Copenhagen, Denmark; 2 Department of Cardiology, Odense University Hospital, Odense, Denmark; 3 Department of Anaesthesiology and Intensive Care, Akershus University Hospital, Lørenskog, Norway; 4 Department of Cardiothoracic Anaesthesia, Copenhagen University Hospital, Rigshospitalet, Copenhagen, Denmark; 5 Department of Clinical Medicine, University of Copenhagen, Copenhagen, Denmark; 6 Department of Cardiothoracic Anaesthesia, Odense University Hospital, Odense, Denmark

**Keywords:** Cardiogenic shock, Acute heart failure, Mechanical circulatory support, Left ventricular assist device, Vasopressor therapy

## Abstract

**Background and aim:**

This study aimed to assess right ventricular (RV) function during cardiogenic shock due to acute left ventricular (LV) failure, including during LV unloading with Impella CP and an added moderate dose of norepinephrine.

**Methods:**

Cardiogenic shock was induced by injecting microspheres in the left main coronary artery in 18 adult Danish Landrace pigs. Conductance catheters were placed in both ventricles and pressure-volume loops were recorded simultaneously.

**Results:**

Cardiogenic shock due to LV failure also impaired RV performance, which was partially restored during haemodynamic support with Impella CP, as demonstrated by changes in the ventriculo-arterial coupling (Ea/Ees ratio) (baseline (median [Q1;Q3]) 1.2 [1.1;1.6]), cardiogenic shock (3.0 [2.4;4.5]), Impella CP (2.1 [1.3;2.7]) (p_Baseline vs CS_ < 0.0001, p_CS vs Impella_ = 0.001)). Impella CP support also improved RV stroke work (SW) (cardiogenic shock 333 [263;530] vs Impella CP (830 [717;1121]) (*p* < 0.001). Moderate norepinephrine infusion concomitant with Impella CP further improved RV SW (Impella CP (818 [751;1065]) vs Impella CP+moderate norepinephrine (1231 [1142;1335]) (*p* = 0.01)) but at the expense of an increase in LV SW (Impella CP (858 [555;1392]) vs Impella CP+moderate norepinephrine (2101 [1024;2613]) (*p* = 0.04)).

**Conclusions:**

The Impella CP provided efficient LV unloading, improved RV function, and end-organ perfusion. Moderate doses of norepinephrine during Impella support further improved RV function, but at the expense of an increase in SW of the failing LV.

## Introduction

Cardiogenic shock is the most severe manifestation of ventricular failure, with 30-day mortality remaining as high as 50% [1–3]. In the attempt to improve the prognosis of the patients with cardiogenic shock, the use of mechanical circulatory support has drastically changed, as the use of axial flow pumps and extracorporeal life support has increased in recent years [4]. The Impella CP, a transvalvular axial flow pump capable of ejecting 3.5 L/min oxygenated blood from the LV to the ascending aorta, is one of the most frequently used devices in cardiogenic shock. However, observational studies assessing the effect of the Impella devices in cardiogenic shock show mixed results and to date, no adequately powered randomized controlled trial has been conducted. Given the complex and emergent nature of cardiogenic shock and subsequent difficulty in conducting controlled trials, other studies assessing different aspects of physiologic changes during treatment with the Impella CP is important to enable optimisation of the devices used. Previous animal studies have shown that the Impella CP is efficient in terms of acute left ventricular (LV) unloading and flow restoration, although organ support is not as efficient as treatment with extracorporeal life support [5].

Cardiac output (CO) is equally provided by the right and left ventricle, and although the two ventricles work in series, there is an interventricular dependency, wherein changes in one ventricle may significantly impact the other. As a result, it is potentially possible to improve the total CO following LV failure by improving right ventricular (RV) function and vice versa. These aspects are largely unexplored, particularly during conditions of use of mechanical circulatory support [6–8].

Therefore, this study aimed to assess RV function in terms of stroke work (SW), pressure-volume area (PVA), and the interventricular end-diastolic volume (EDV) relationship during experimentally induced profound cardiogenic shock in pigs caused by microsphere injections in the left main coronary artery, leading to LV failure. Furthermore, to evaluate the effect of additional moderate infusions of norepinephrine on cardiac function during LV unloading with the Impella CP.

## Methods

### Animals, setup, and instrumentation

The present study pooled data from two consecutive series of experiments performed by the same research group [5, 9, 10]. A total of 18 Danish female Landrace pigs weighing 70–75 kg were studied. All experiments were approved by the Danish animal experiments inspectorate (ID number: 2016-15-00951). The experimental setup and method of cardiogenic shock induction was consistent in all the included animals and has previously been described in detail [9]. Briefly, animals were initially anaesthetised and mechanically ventilated. All sheaths for instrumentation were placed using the Seldingers technique. The following instruments were inserted via the sheaths: (1) Conductance catheters (Ventri-Cath 512 pressure-volume Loop Catheter, Millar Inc. Houston, USA) were placed in the RV and LV for pressure-volume measurements and an additional one was placed retrogradely in the descending aorta to monitor aortic pressure, (2) a combo Swan Ganz (Edwards Lifesciences Corp. Irvine, USA) was placed in the pulmonary artery to continuously measure CO and central mixed saturation (SvO2), (3) a JL 3.5 guide catheter (Launcher; Medtronic, Minneapolis, USA) was placed in the left main coronary artery for microsphere injections, (4) and the Impella CP was inserted through an arterial access in the groin. The same regimen of 1000 mL isotonic saline/hour was administered to minimise the bias of fluid treatment.

Cardiogenic shock was induced by a stepwise injection of a solution containing 0.125 g polyvinyl alcohol microspheres (Contour™; Boston Scientific, Marlborough, USA) dissolved in 10 mL saline and 10 mL contrast in the left main coronary artery. Profound cardiogenic shock was defined as an at least 50% reduction of SvO2 compared to baseline or absolute SvO2 below 30% and/or a sustained CO ≤ 2.0 L/min.

### Experimental protocol

Following the induction of cardiogenic shock, an Impella CP was inserted through the femoral artery and placed across the aortic valve using fluoroscopic guidance. Throughout the study, the Impella CP was running on the highest performance level possible, in order to avoid suction events. A supplement of norepinephrine was administered if the mean arterial pressure (MAP) decreased below 45 mmHg to maintain an adequate perfusion pressure. In 8 pigs, the norepinephrine dose was increased with 0.10 μg/kg/min per protocol following treatment with 30 min of Impella CP and a minimum dose of norepinephrine.

### RV and LV pressure-volume measurements

The two conductance catheters were inserted under fluoroscopic guidance into the RV and LV via the left external jugular vein and the right carotid artery, respectively. They were connected to a PowerLab 16/35 (ADInstruments, Dunedin, New Zealand) via an MPVS Ultra® pressure-volume loop system (Millar inc. Houston, USA). Pressure-volume loops of the RV and LV were simultaneously and continuously recorded in LabChart Pro (ADInstruments, Dunedin, New Zealand). Volume calibration was done using an alpha correctional value, and parallel wall conductance was estimated using the hypertonic saline method. A baseline preload occlusion of the inferior vena cava at the level of the diaphragm was performed with a balloon occlusion catheter (Nucleus, NuMED, Cornwall, On Canada) in all animals to estimate the theoretical volume where zero pressure is generated (Vo). Vo was used as the constant towards the calculation of the pressure-volume area (PVA, mmHg × mL) and end-systolic pressure-volume relationship (Ees, mmHg/mL). Furthermore, the following physiologic measures were obtained in both the RV and LV; EDV (mL) end-diastolic pressure (EDP, mmHg), end-systolic pressure (ESP mmHg), SW (mmHg × mL), potential energy (mmHg × mL), arterial elastance (Ea, mmHg/mL), and the ventriculo-arterial coupling calculated as Ees/Ea.

### Data collection

Haemodynamic parameters including MAP, heart rate (HR), pulmonary artery pressure (PAP), central venous pressure (CVP), and CO were collected at baseline and every 15 min throughout the study. Pressure-volume parameters were analysed at the following timepoints, including (1) at baseline before microsphere injection, (2) after induction of cardiogenic shock (prior to intervention), (3) after 30 min of Impella CP intervention. An additional set of measurements were collected after 30 min of combination therapy with Impella CP and a moderate infusion dose of norepinephrine in the 8 pigs receiving a dose escalation of norepinephrine.

### Statistics

Data with normal distribution are presented as mean (standard deviation, SD), and non-normal distribution is presented as median [Q1, Q3]. To assess the difference in the variables over time, a repeated *t* test or signed-rank test was used as appropriate, with Bonferroni post hoc adjustment of the *p* value. Statistical analyses were performed with STATA IC15 (StataCorp, Texas, USA). A *p* value ≤0.05 was considered statistically significant.

## Results

### Effect of cardiogenic shock induction

**Fig. 1:**
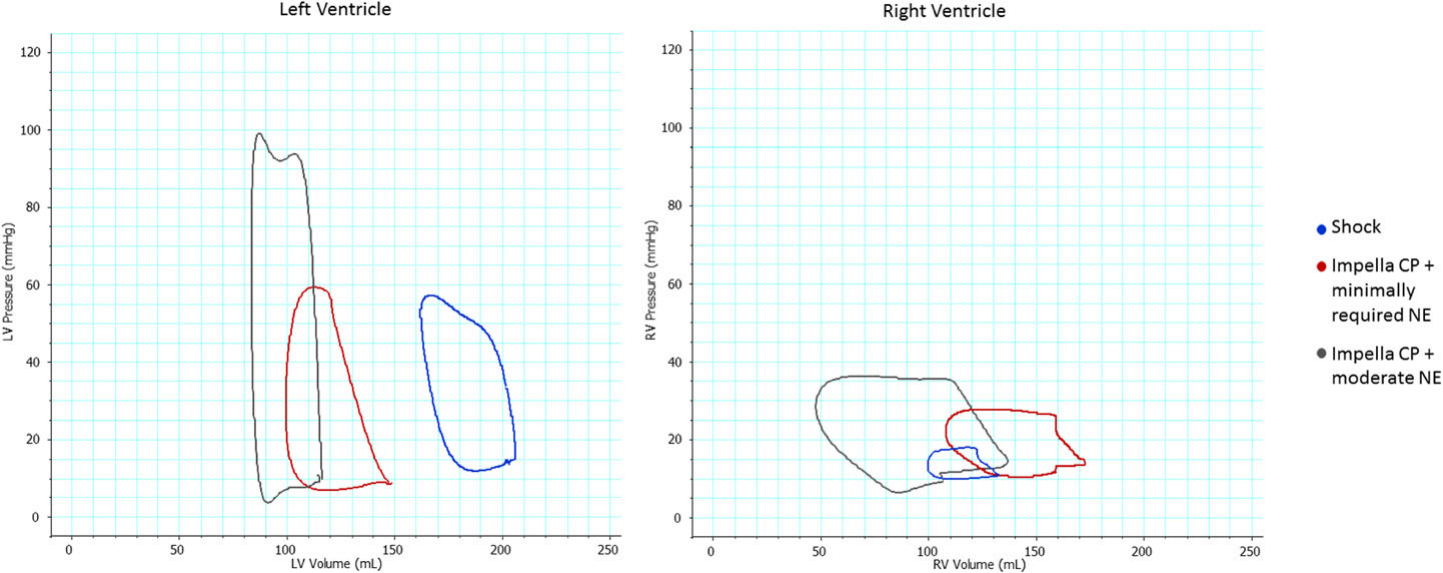
Left and right ventricular pressure-volume loops during shock (blue loops), Impella CP, and minimally required dose of norepinephrine (red loops) and Impella CP + moderate dose of norepinephrine (gray loops). LV: left ventricle, NE: norepinephrine, RV: right ventricle

Profound cardiogenic shock was achieved in all the pigs following repetitive injections of microspheres in the left main coronary artery, causing a significant reduction in CO, SvO_2_ and MAP (Table 1). The induction of cardiogenic shock was associated with backward failure, demonstrated by a significant increase in the diastolic PAP, CVP (Table 1), and RV Ea (Table 2). The LV was dilated and strained, as shown by a significant increase in LVEDV and LVEDP (Fig. 1, Table 2). Left ventriculo-arterial decoupling was evident with a four-fold increase in the LV Ea/Ees ratio (Table 2). Dilatation of the LV significantly reduced the EDV ratio between the two ventricles (Fig. 1), and right ventriculo-arterial decoupling was also evident with a doubling of the RV Ea/Ees ratio (Table 2). SW was significantly reduced in both ventricles, whereas potential energy increased in the RV but decreased in the LV due to the marked reduction in LVESP (Table 3). The ratio of RV/LV SW and RV/LV total ventricular work (PVA × HR) showed a trend towards increase, although not statistically significant (Table 3).

**Table 1:** Haemodynamics in profound CS treated with Impella CP

	Baseline, *n* = 18	Shock, *n* = 18	Impella CP+minimally required NE, *n* = 18	*p* value
NE dose, μg/kg/min, median (Q1, Q3)	0.00 (0.0, 0.04)	0.05 (0.00, 0.06)	0.05 (0.02, 0.10)	
SvO2, %, mean (SD)	72 (9)	33 (8)	57 (12)	<0.001 for all
Cardiac output, L/min, mean (SD)	5.5 (0.9)	2.9 (1.1)	4.9 (1.2)	Base vs shock: <0.001 Shock vs Imp: <0.001
MAP, mmHg, mean (SD)	71 (13)	39 (10)	60 (11)	Base vs shock: <0.001 Shock vs Imp: <0.001
Heart rate, bpm, mean (SD)	75 (10)	74 (10)	78 (9)	Base vs shock: 1.00 Shock vs Imp: 0.33
PAPsystolic, mmHg, mean (SD)	28 (4)	30 (6)	32 (6)	Base vs shock: 0.52 Shock vs Imp: 0.30
PAPdiastolic, mmHg, mean (SD)	14 (5)	19 (5)	18 (5)	Base vs shock: <0.001 Shock vs Imp: 1.00
PAPmean, mmHg, mean (SD)	21 (4)	24 (6)	25 (5)	Base vs shock: 0.005 Shock vs Imp: 1.00
CVP, mmHg, mean (SD)	8 (3)	12 (4)	10 (4)	Base vs shock: <0.001 Shock vs Imp: 0.005

*NE* norepinephrine, *MAP* mean arterial pressure, *PAP* pulmonary artery pressure, *CVP* central venous pressure

**Table 2:** Pressure-volume in RV and LV during CS and mechanical support

	Baseline, *n* = 18	Shock, *n* = 18	Impella CP + minimally required NE, *n* = 18	*p* value
RV EDV, mL, mean (SD)	173 (28)	169 (41)	190 (34)	Base vs shock: 1.00 Shock vs Imp: 0.07
RV ESP, mmHg, median (Q1, Q3)	26 (25, 28)	27 (25, 30)	29 (27, 34)	Base vs shock: 1.00 Shock vs Imp: 0.006
RV EDP, mmHg, mean (SD)	11 (3)	13 (3)	13 (3)	Base vs shock: 0.001 Shock vs Imp: 0.73
RV Ees, mmHg/mL, median (Q1, Q3)	0.29 (0.22, 0.34)	0.24 (0.19, 0.29)	0.25 (0.18, 0.36)	Base vs shock: 0.07 Shock vs Imp: 0.30
RV Ea, mmHg/mL, median (Q1, Q3)	0.38 (0.31, 0.40)	0.68 (0.64, 0.78)	0.45 (0.40, 0.55)	Base vs shock: <0.001 Shock vs Imp: 0.009
RV Ea/Ees ratio, median (Q1, Q3)	1.2 (1.1, 1.6)	3.0 (2.4, 4.5)	2.1 (1.3, 2.7)	Base vs shock: <0.001 Shock vs Imp: 0.001
LV EDV, mL, mean (SD)	196 (25)	254 (34)	162 (41)	Base vs shock: <0.001 Shock vs Imp: <0.001
LV ESP, mmHg, median (Q1, Q3)	91 (84, 99)	56 (45, 62)	62 (56, 75)	Base vs shock: <0.001 Shock vs Imp: 0.07
LV EDP, mmHg, mean (SD)	14 (3)	20 (4)	15 (5)	Base vs shock: <0.001 Shock vs Imp: <0.001
LV Ees, mmHg/mL, median (Q1, Q3)	0.83 (0.66, 1.02)	0.27 (0.24, 0.32)	0.56 (0.45, 0.73)	Base vs shock: <0.001 Shock vs Imp: <0.001
LV Ea, mmHg/mL, median (Q1, Q3)	1.2 (1.13, 1.45)	1.59 (1.32, 1.97)	2.05 (0.39, 2.74)	Base vs shock: 0.29 Shock vs Imp: 0.04
LV Ea/Ees, median (Q1, Q3)	1.5 (1.3, 1.9)	6.5 (5.0, 8.2)	3.8 (2.1, 4.8)	Base vs shock: <0.001 Shock vs Imp:<0.001

*NE* norepinephrine, *RV* right ventricle, *LV* left ventricle, *EDV* end-diastolic volume, *ESP* end-systolic pressure, *EDP* end-diastolic pressure, *Ees* end-systolic pressure-volume relationship, *Ea* arterial elastance

**Table 3:** Cardiac work in RV and LV during CS and mechanical support

	Baseline, *n* = 18	Shock, *n* = 18	Impella CP + minimally required NE, *n* = 18	*p* value
RV SW, mmHg × mL, median (Q1, Q3)	1060 (874, 1287)	333 (263, 530)	830 (717, 1121)	Base vs shock: <0.001 Shock vs Imp: <0.001
RV PE, mmHg × mL, mean (SD)	1.242 (375)	1.662 (529)	1.835 (612)	Base vs shock: 0.001 Shock vs Imp: 0.34
RV PVA × HR*10^3^, mmHg/min, mean (SD)	177 (40)	155 (50)	215 (44)	Base vs shock: 0.171 Shock vs Imp<0.001
LV SW, mmHg × mL, mean (SD)	4351 (4009, 5367)	845 (587, 1421)	1057 (595 1725)	Base vs shock: <0.001 Shock vs Imp: 1.00
LV PE, mmHg × mL, mean (SD)	7986 (1007, 10575)	6495 (5460, 8060)	4714 (3752, 5964)	Base vs shock: 0.004 Shock vs Imp: 0.008
LV PVA × HR*10^3^, mmHg/min, mean (SD)	751 (182)	497 (164)	399 (173)	Base vs shock: <0.001 Shock vs Imp: 0.15
RV/LV PVA × HR ratio, median (Q1, Q3)	0.24 (0.22, 0.28)	0.30 (0.26, 0.38)	0.53 (0.41, 0.76)	Base vs shock: 1.00 Shock vs Imp: 0.001
RV/LV ratio of SW, median (Q1, Q3)	0.23 (0.20, 0.28)	0.44 (0.31, 0.63)	0.82 (0.52, 1.60)	Base vs shock: 0.06 Shock vs Imp: 0.08

NE norepinephrine, *RV* right ventricle, *LV* left ventricle, *SW* stroke work, *PE* potential energy, *PVA* pressure-volume area, *HR* heart rate

### Effect of Impella CP

**Fig. 2:**
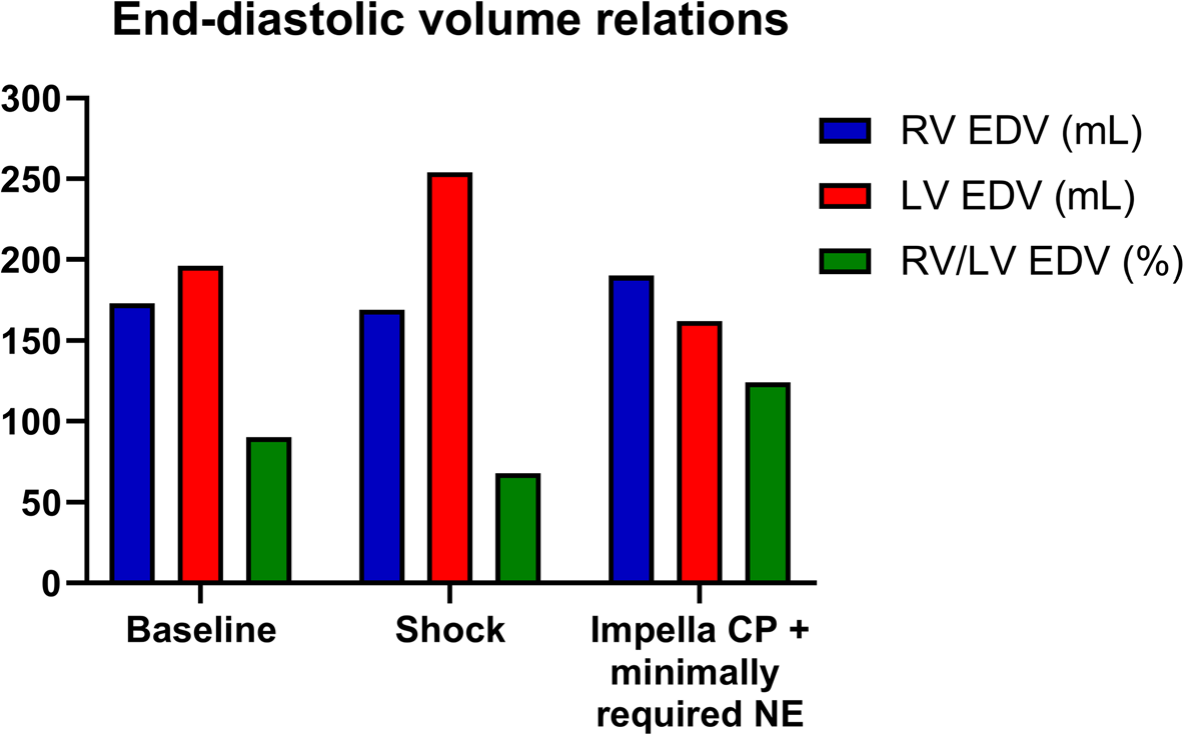
Bar chart depicting end-diastolic volume relations between left and right ventricle at baseline, shock, and during Impella CP + minimum norepinephrine dose. EDV: end-diastolic volume, LV: left ventricle, NE: norepinephrine, RV: right ventricle

Despite an improvement in flow with increased CO and SvO_2_ after initiation of Impella CP support, a minimum dose of norepinephrine was required in 16 out of 18 animals to maintain a MAP >45 mmHg, Table 1. During Impella CP support, the CVP decreased, but the diastolic PAP remained unchanged. The volume unloading of the LV was evident from the significant reduction in LVEDV and LVEDP (Table 2). In contrast to the leftward shift of the LV pressure-volume loop, the RV dilated leading to a rightward shift of the pressure-volume loop (Fig. 1) and consequently the inversion of the RV/LV EDV volume ratio (Fig. 2). The RV dilatation enabled the generation of a higher RVESP, and consequently, the RV afterload (Ea) decreased (Table 2). Following the initiation of Impella CP + minimally required norepinephrine, both ventricles improved their ventriculo-arterial coupling (Table 2). Also, the RV SW more than doubled (*p* < 0.001), whereas SW remained unchanged in the LV. Impella CP support significantly reduced the potential energy in the LV, but not in the RV. Therefore, the ratio of RV/LV total cardiac work (PVA × HR) increased significantly due to combined changes in SW and potential energy (Table 2).

### Effect of increased norepinephrine

Following 30 min of Impella CP support, the norepinephrine infusion was increased with 0.1 μg/kg/min per protocol in 8 animals, leading to a further increase in CO and SvO_2_, but not MAP (Table 4). An increase in HR mainly drove the increase in CO. The CVP decreased further, but pulmonary pressures increased significantly, which was partly related to an increase in RV contractility and the ability to generate higher pressure, as seen by the increase in RVESP (Table 5). The RV Ea also showed a trend towards increase. The increase in norepinephrine dose resulted in an increased SW in both ventricles, in contrast to Impella CP support, which led to an increase in SW only in the RV.

**Table 4:** Haemodynamics during Impella CP going from minimum to moderate norepinephrine

	Impella CP+minimally required NE, *n* = 8	Impella CP + moderate NE, *n* = 8	*p* value
NE dose, μg/kg/min, median (range)	0.03 (0.00–0.10)	0.1 (0.10–0.18)	
Svo2, %, mean (SD)	54 (12)	70 (15)	<0.001
Cardiac output, L/min, mean (SD)	4.4 (0.9)	5.5 (0.9)	0.01
MAP, mmHg, median (Q1, Q3)	61 (58, 63)	64 (60, 75)	0.08
Heart rate, bpm, median (Q1, Q3)	77 (70, 82)	98 (89, 111)	0.01
PAPsystolic, mmHg, median (Q1, Q3)	31 (24, 36)	42 (37, 43)	0.01
PAPdiastolic, mmHg, mean (SD)	19 (4)	22 (6)	0.03
PAPmean, mmHg, median (Q1, Q3)	25 (20, 29)	30 (29, 34)	0.01
CVP, mmHg, mean (SD)	12 (4)	11 (4)	0.04

*NE* norepinephrine, *MAP* mean arterial pressure, *PAP* pulmonary artery pressure, *CVP* central venous pressure

**Table 5:** Pressure-volume relationship and cardiac work in RV and LV with increased dose of norepinephrine

	Impella CP+minimally required NE, *n* = 8	Impella CP+moderate NE, *n* = 8	*p* value
**Pressure-volume relationship**			
RV EDV, mL, mean (SD)	186 (30)	151 (18)	0.03
RV ESP, mmHg, median (Q1, Q3)	28 (27, 32)	37 (34, 41)	0.03
RV EDP, mmHg, mean (SD)	13 (2)	11 (3)	0.01
RV Ees, mmHg/mL, median (Q1, Q3)	0.22 (0.18, 0.35)	0.42 (0.35, 0.49)	0.03
RV Ea, mmHg/mL, median (Q1, Q3)	0.52 (0.42, 0.54)	0.67 (0.59, 0.81)	0.09
RV Ea/Ees ratio, median (Q1, Q3)	2.1 (1.7, 2.7)	1.5 (1.3, 2.2)	0.12
LV EDV, mL, mean (SD)	171 (42)	168 (42)	0.90
LV ESP, mmHg, median (Q1, Q3)	60 (57, 64)	85 (84, 88)	0.02
LV EDP, mmHg, mean (SD)	16 (5)	16 (5)	0.77
RV/LV EDV ratio, median (Q1, Q3)	1.14 (0.85, 1.34)	0.87 (0,72, 1.19)	0.12
RV/LV EDP ratio, median (Q1, Q3)	0.91 (0.69, 1.08)	0.73 (0.54, 0.96)	0.11
LV Ees, mmHg/mL, median (Q1, Q3)	0.50 (0.40, 0.62)	0.78 (0.59, 0.87)	0.04
LV Ea, mmHg/mL, median (Q1, Q3)	2.05 (1.49, 3.42)	2.49 (1.84, 3.14)	0.48
LV Ea/Ees, median (Q1, Q3)	4.3 (3.8, 6.2)	3.1 (2.6, 4.7)	0.12
**Cardiac work**			
RV SW, mmHg × mL, median (Q1, Q3)	818 (751, 1.065)	1.231 (1.142, 1.335)	0.01
RV PE, mmHg × mL, mean (SD)	1.787 (291)	1.707 (469)	0.63
RV PVA × HR*10^3^, mmHg/min, mean (SD)	205 (27)	299 (77)	0.003
LV SW mmHg × mL, median (Q1, Q3)	858 (555, 1.392)	2.101 (1.024, 2.613)	0.04
LV PE mmHg × mL, mean (SD)	3.810 (1.105)	4.833 (1.695)	0.28
LV PVA × HR*10^3^, mean (SD) mmHg/min	363 (89)	687 (235)	0.007

*NE* norepinephrine, *RV* right ventricle, *LV* left ventricle, *EDV* end-diastolic volume, *ESP* end-systolic pressure, *EDP* end-diastolic pressure, *Ees* end-systolic pressure-volume relationship, *Ea* arterial elastance, *SW* stroke work, *PE* potential energy, *PVA* pressure-volume area, *HR* heart rate

## Discussion

The induction of cardiogenic shock by injecting microspheres in the LM caused profound LV failure but the RV performance was also severely impaired, as demonstrated by ventriculo-arterial decoupling in both ventricles. The initiation of Impella CP support improved haemodynamics and unloaded the LV, in terms of a higher CO and SvO_2_, and a reduction in LVEDV and LVEDP. Due to the ventricular interdependency within the pericardial constraint, unloading of the LV consequently allowed better filling of the RV with a complete reversion of the RV/LV EDV ratio. This permitted higher RV pressure generation and consequently led to an increase solemnly in the RV SW. An additional moderate norepinephrine dose improved CO and SvO_2_ further; however, this effect came at the cost of increased SW in the failing LV.

The main goal of cardiogenic shock treatment is to re-establish end-organ perfusion. Moreover, recently, unloading of the failing ventricle has gained importance as studies have shown a beneficial effect on infarct size reduction [11]. In the present study, cardiogenic shock due to LV failure significantly impaired RV performance. This may partly be explained by the increased afterload caused by LV backward failure. In addition, dilatation of the LV compromises RV filling with significantly increased RV-EDP and consequently increased ventriculo-arterial decoupling of the RV, which is continuously balanced as the relationship between contractility and afterload. In accordance with the current study, Pagnamenta et al. also found concomitant RV impairment and only a slight increase in PAP pressures, when experimentally inducing chronic heart failure in dogs, which emphasises the importance of RV contractility and consequently ventriculo-arterial coupling in both acute and chronic heart failure [12–14]. Previous studies have reported that under normal circumstances, 20–40% of RV pressure and outflow are generated by septal power generation in the LV [15, 16]. Conversely, the present study suggests that acute LV failure impairs RV function. Consequently, RV impairment will lead to reduced LV filling and thereby perpetuate the vicious cycle of cardiogenic shock [17].

Treatment with Impella CP and the minimally required dose of norepinephrine to maintain an adequate coronary perfusion pressure fulfilled the two main aims in the treatment of cardiogenic shock, namely LV unloading and an increase in vital organ perfusion in terms of MAP, CO, and SvO2. In line with existing clinical studies, most animals required a minimal supplementary infusion of norepinephrine to maintain an adequate coronary, cerebral, and renal perfusion pressure when treated with Impella CP [18, 19]. The Impella CP can deliver up to 3.5 L/min, which may be insufficient in patients with a larger body surface area or those with profound cardiogenic shock and complete dependency on mechanical support for CO .

In clinical experience, inadequate filling of the LV during Impella CP support leads to LV suction events, resulting in a decrease in output from the device and increased risk of haemolysis. In this situation, optimisation of Impella placement is crucial and flow may further be augmented by optimising RV performance and consequently LV filling. Unloading of the LV caused a left-sided septal shift within the pericardial constraint, resulting in improved RV function evident by a 2.5-fold increase in RV SW and an increased CO despite an unchanged LV SW.

Compared to the stage of shock the interventions of the current study either resulted in increased or unaltered pulmonary pressures thus a very different pathophysiological situation as RV dysfunction due to RV pressure overload or chronic LV failure with postcapillary increase in pulmonary artery pressure. There are no robust studies regarding inodilators in the treatment of cardiogenic shock, but the results of the current study suggest that pulmonary vasodilators could theoretically be beneficial in restoring CO during Impella CP treatment [20]. In a pressure RV overload canine model Kerbaul et al. demonstrated an increase in CO and Ees with moderate and high dose of norepinephrine but to a lesser degree than dobutamine [21]. However, due to fundamentally different causes for RV failure, extrapolation of these results to the situation with profound systemic hypotension and CO should be done with great caution. In the present study, a moderate dose of norepinephrine (0.10 μg/kg/min) led to an unbalanced increase in SW of the RV by 150% and LV by 250%. Consequently, the RV/LV SW ratio decreased from 0.95 to 0.58. Previous animal studies have demonstrated a correlation between SW and infarct size in the failing heart. Therefore, although speculative, the increase in SW of the failing LV could have a negative impact and may affect infarct healing and ultimately outcome [22].

## Limitations

The present study has several limitations. A minimum dose of norepinephrine and Impella CP had to be initiated simultaneously. Therefore, the isolated effect of each intervention cannot be teased out, and haemodynamic data on the isolated effect of each intervention would have been optimal. However, considering the severity of the cardiogenic shock, the animals were often on the verge of cardiac collapse, which necessitated immediate mechanical circulatory support. From pilot studies, we experienced that if the MAP fell below 45 mmHg, the animals were quite prone to develop ventricular fibrillation, and for this reason, a minimal dose of norepinephrine was most often required. Given the limited number of experimental pigs, the risk of a type 2 error cannot be excluded in the comparisons of haemodynamic and conductance derived variables. Finally, despite the similarity in size and anatomy, there may be species-specific differences in the effect of norepinephrine and Impella support in young Danish Landrace pigs with highly compliant arterial systems compared with the more elderly human population suffering from cardiogenic shock.

## Conclusion

In this large animal model of profound cardiogenic shock due to acute LV failure, RV function was also significantly affected as a result of the displacement of the interventricular septum. Treatment with Impella CP and a minimum dose of norepinephrine provided efficient unloading of the failing LV, improved RV function, and end-organ perfusion. An additional increase in the dose of norepinephrine during Impella CP support further increased CO and improved RV function. However, the increase in norepinephrine dose also increased the SW of the failing LV. These results indicate a likely trade-off point, wherein increasing doses of norepinephrine infusion improve RV function and end-organ perfusion at the expense of increased energy expenditure (PVA) of the failing LV.

## Data Availability

The datasets used and/or analysed during the current study are available from the corresponding author on reasonable request.

## Abbreviations

CO: Cardiac output
CVP: Central venous pressure
EDV: End-diastolic volume
EDP: End-diastolic pressure
ESP: End-systolic pressure
LV: Left ventricle
MAP: Mean arterial pressure
PAP: Pulmonary artery pressure
PVA: Pressure-volume area
RV: Right ventricle
SW: Stroke work
